# Deep-Learning-Driven Dual Sensors Based on Single-Trapped Upconverting Particles

**DOI:** 10.1021/acs.nanolett.6c03652

**Published:** 2026-10-20

**Authors:** Óscar Pérez-Benito, J. Ignacio Echavarría, Javier Hernandez-Rueda

**Affiliations:** † IMDEA Nanociencia, Calle Faraday 9, 28049 Madrid, Spain; ¶ Instituto de Química Física Blas Cabrera, IQF-CSIC, Serrano 119, 28006 Madrid, Spain; ‡ Optics Department, Faculty of Physics, 16734 University Complutense of Madrid. Plaza de Ciencias 1, 28040 Madrid, Spain

**Keywords:** sensors, deep learning, upconverting particles, levitating particles, nonlinear optics, Paul trap, luminescence

## Abstract

Upconverting lanthanide-doped particles are widely used as nanoscale temperature sensors, particularly in biological and photonic environments. However, extending their functionality to simultaneously measure temperature and light intensity remains a challenge, as the luminescent response is highly sensitive to both the environment composition and its temperature. In this work, we combine deep-learning methods with large-scale spectral data sets to investigate the reliability of Er/Yb-doped particles to function as dual sensors. Spectral data are collected from single particles levitating in vacuum and ensembles of particles in application-relevant wet and dry media. Although traditional ratiometric methods reliably retrieve temperature irrespective of the environment and specific experimental apparatus, they fail to disentangle the complex dependencies needed for dual sensing. Our trained models overcome these limitations, accurately predicting both temperature and laser power despite environmental variability. This approach opens a new route toward robust, data-driven sensing with upconverting particles in complex or fluctuating conditions.

## Graphical abstract

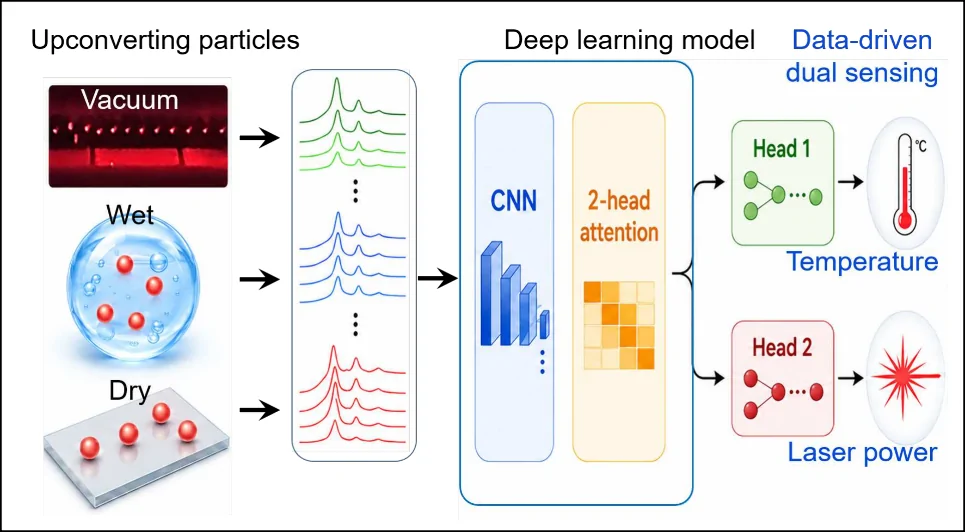

Today, luminescent nanoparticles, mainly lanthanide-doped upconverting nanoparticles (UCNPs), are consolidated as powerful tools for a variety of sensing applications, including biosensing, remote sensing, and environmental monitoring.^1,2^ These nanoparticles offer remarkable advantages, such as minimal phototoxicity, outstanding chemical- and photostability, sharp emission bands, and large quantum yields.^3,4^ A fundamental aspect employed in luminescence-based sensing, especially nanothermometry, is the utilization of emission intensity ratios from distinct electronic transitions, enabling precise and robust nanoscale temperature measurements.^5^ Specifically, Er/Yb-doped UCNPs exploit the Boltzmann distribution of excited electronic states to accurately determine temperature through spectral peak ratios, a method widely validated across various application contexts.^6,7^ Furthermore, recent advances have made it possible to tailor emission properties of UCNPs, significantly broadening their potential use in diverse applications, from complex biological systems to advanced optoelectronic devices.^8,9^

In recent years, research has increasingly focused on the use of lanthanide-based nanoparticles as dual sensors capable of simultaneously measuring temperature and laser power.^5^ Although their reliability as temperature sensors has been robustly established over the past decade, their application as laser power sensors, though recently explored, is compromised by environmental conditions (i.e., chemical and physical properties of the surrounding medium) and temperature-dependent variations in luminescence yield, undermining their utilization as dual sensors. Various research groups have extensively tested several environmental factors that affect the luminescent response of UCNPs, further highlighting this complexity.^10,11^ In a recent work, our group compared the intrinsic optical behavior of single Er/Yb-doped particles trapped in a vacuum with the aggregate response of ensembles of particles in wet and dry media, highlighting how temperature influences key transitions that can serve as potential laser-power probes.^12^ In addition to the particle-environment response, the relative luminescence peak intensities across different UV-IR transitions is also intricately influenced by the spectral response of optics and devices within experimental systems, making accurate power sensing particularly challenging. Consequently, ratiometric measurements used for laser-power retrieval are further compromised by their widely spaced UV-IR transitions, which suffer from the aforementioned uneven spectral response in spectroscopy systems.^13^ In contrast, the use of closely spaced green-band transitions for either temperature (i.e., 525 and 546 nm) or laser-power (i.e., 557 nm) retrieval may require multi-Gaussian analysis to fully decouple the contributions of individual transitions to luminescence bands, which further hinders the reliability of UCNPs to work as dual sensors.^5^ This specific issue becomes particularly relevant at high laser powers, where three-photon-assisted mechanisms contribute significantly to the 557 nm spectral peak. The inherent limitations of UCNPs highlight the need for alternative methods that complement ratiometric thermometry and broaden their application as dual sensors. More broadly, data-driven and multivariate approaches have recently demonstrated substantial improvements in luminescence thermometry by exploiting information distributed across the full emission spectrum. These include support-vector-machine-based thermometry,^14^ chemometric regression methods,^15^ automated machine-learning approaches,^16^ and full-spectrum random-forest and Gaussian-process regression.^17^ These studies demonstrate the potential of full-spectrum analysis for single-parameter thermometry. However, its extension to the simultaneous retrieval of temperature and laser power, particularly across different particle environments, remains largely unexplored.

In this Letter, we present a novel approach that integrates automated large-scale spectral data collection with deep learning models, allowing the use of UCNPs as dual sensors of temperature and laser power. We systematically measure and analyze the luminescent response of Er/Yb-doped particles in three experimental configurations: ensembles of particles on a substrate in air, ensembles in water, and single particles levitating in vacuum. For the latter configuration, we use a quadrupolar Paul trap to isolate the intrinsic luminescence response of single particles, offering a unique experimental approach compared with conventional ensemble-based methods and a technically challenging environment in which to train and validate the robustness of our AI models. The data sets span broad temperature ranges and laser powers, providing an extensive basis for training neural network models. Our deep-learning architecture, based on convolutional neural networks, successfully unravel the complex interdependencies among temperature, laser power, and environmental conditions. Consequently, this data-driven methodology reliably enables dual-mode sensing, effectively addressing previous limitations, and providing better accuracy in complex and dynamic environmental scenarios.

We systematically collected large spectral data sets emitted by NaY_0.77_Yb_0.2_Er_0.03_F_4_ microspheres (756555-25G, Sigma-Aldrich) using the automated spectroscopy setup sketched in Figure 1a.^12,18^ The experiments were first performed on ensembles of particles deposited on a substrate (borosilicate glass slide) and in water solution and subsequently on single trapped particles. The spectra were acquired for laser intensities in the 10^–1^-10^4^ W/cm^2^ range and temperatures between 10 and 70 °C, providing approximately 5 × 10^4^ spectra per investigated environment. Laser excitation was automated using a laser current controller and a continuous-wave diode laser at 980 nm. The laser was focused on the luminescent samples using a microscope objective (Mitutoyo, Plan-Apochromat, 10×, NA = 0.28). The emission was collected by the same objective and routed to a fiber-based spectrometer. Temperature control and real-time monitoring were achieved using a thermal bath circulator and a calibrated thermocouple with a multimeter, respectively. Single-particle experiments were conducted using a quadrupole Paul trap inside a transparent vacuum chamber.^12,19,20^ We employed an ethanol-based suspension and an electrospray emitter (Taylor cone at 2.9 kV^21^) to separate and introduce single particles into the trap. The trap generates a time-dependent quadrupole potential, enabling stable trapping under pressures down to 10^–3^ mbar (see more details in the Supporting Information). Figure 1b illustrates the sample configurations used for particle ensembles on a substrate and in water, together with an example image of luminescent particles confined in the Paul trap.

**Figure 1:**
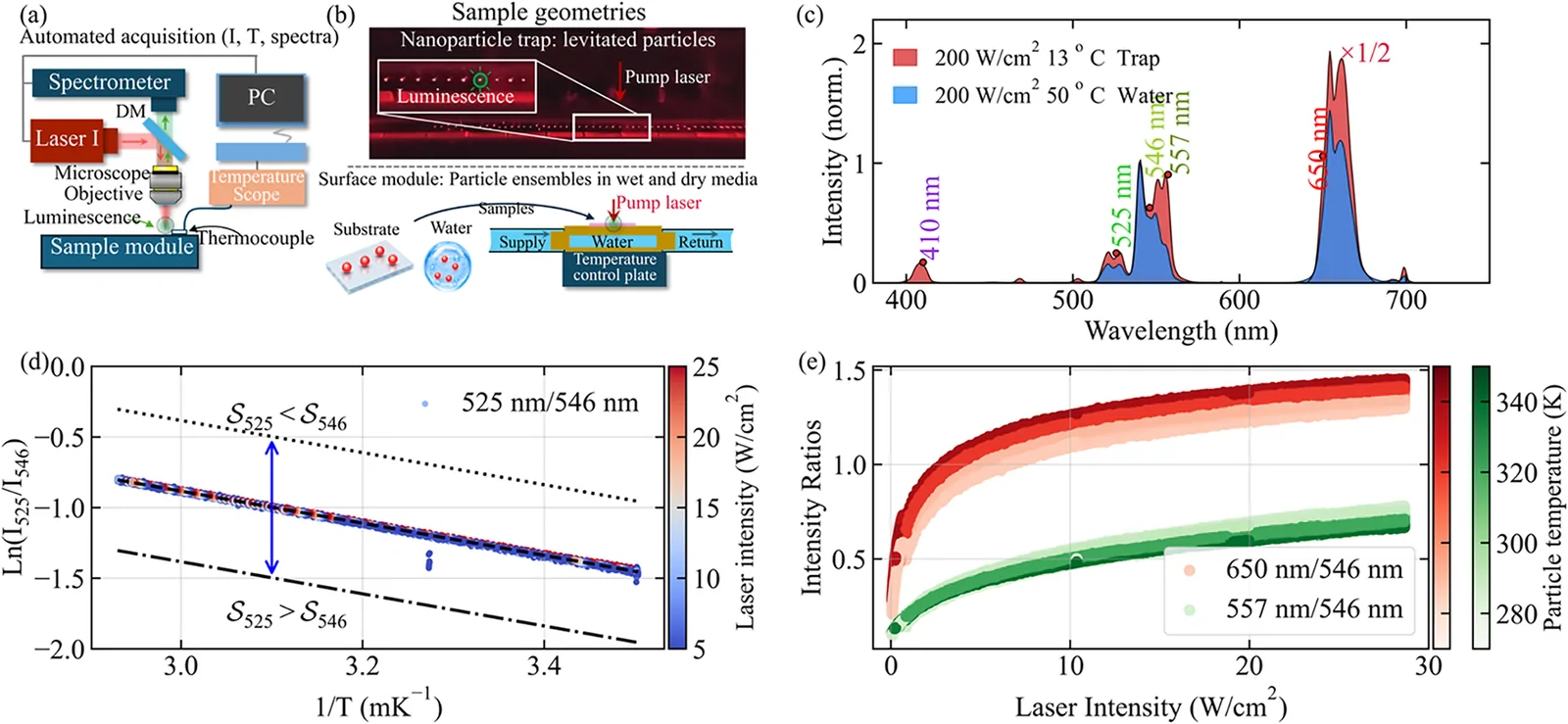
(a) Schematic of the automated spectroscopy setup for large data acquisition. (b) Sample geometries, including the ensemble surface module and the trap system. The bright-field imageshowslevitating upconversion particles inside trap system under simultaneous 980 nm excitation and 642 nm probe illumination. (c) Emission spectra of a single-trapped particle (red) and particles embedded in water (blue) under 980 nm excitation, showing characteristic Er^3+^ emission bands at 410, 525, 546, 557, and 650 nm. (d) Graph of the ratiometric data (525 nm/546 nm) versus temperature for several laser intensities (color scale). (e) Graph of both the 650 nm/546 nm (red) and 557 nm/546 nm (green) intensity ratios as a function of laser intensityfor a large range of temperatures.

Figure 1c shows two spectra acquired using the same laser excitation at 200 W/cm^2^ on single particles (red) and on ensembles of particles in water (blue). This comparison clearly illustrates the effect of the surrounding environment on the intrinsic luminescent response of Er/Yb-doped particles, which diminishes the efficiency of electronic transitions linked to spectral peaks at 410, 557, and 650 nm (i.e., these peaks are more intense in vacuum, see ref^12^). Additionally, the fact that particles are isolated in a vacuum results in poor heat dissipation and laser-induced heating (e.g., 50 °C at 200 W/cm^2^). The temperature *T* can be retrieved by using the ratio R between the luminescence peaks at 525 nm (*I*_525_) and 546 nm (*I*_546_).^22,23^ These emission peaks originate through the transition from ^2^H_11/2_ and ^4^S_3/2_ excited states to the ground state of Er^3+^. Because the energy gap Δ*E* between these excited states is on the order of a few hundred wavenumbers, the ratio of their populations in thermal equilibrium is dictated by the Boltzmann factor:

1 $$R=\frac{S_{525}I_{525}}{S_{546}I_{546}}=Ae^{-\Delta E/k_{B}T}$$

where *A* is a material-dependent constant^22–24^ and $S_{\lambda }$ is the spectral calibration of the transmission function of the experimental setup (Figure S3). The linearization of this expression illustrates how both *A* and $S_{525}/S_{546}$ contribute to a constant factor $A^{\prime }$ that vertically shifts the ln­(*I*_525_/*I*_546_) versus *T* ^–1^ relationship.

2 $$\ln\left(\frac{I_{525}}{I_{546}}\right)=-\frac{\Delta E}{k_{B}}T^{-1}+\left[\ln\left(A\right)-\ln\left(\frac{S_{525}}{S_{546}}\right)\right]=-mT^{-1}+A^{\prime }$$

Figure 1d presents the ln­(*I*_525_/*I*_546_) – *T* ^–1^ calibration in water where we illustrate the aforementioned calibration shift linked to $A^{\prime }$ with two dashed lines parallel to the experimental calibration. We find a slope of *m* = −1.09 ± 0.02 × 10^3^ K that is in agreement with other values reported in the literature.^12,23^ Our fitted slope value is also in good correspondence with the theoretical value, calculated with the energy gap between the thermally coupled excited states Δ*E*. Taking into account a value of 91 meV, we find a slope of -Δ*E*/*k*_B_ = −1.06 × 10^3^ K^1^.^12^ Our data align with the well-established consensus in the literature that luminescent thermometry remains reliable regardless of laser power or the medium in which the particles are embedded. In contrast, Figure 1e shows that ratiometric-based laser power measurements are compromised by variations in temperature and embedding environment (i.e., the same ratio may lead to different power values).^5,12^ The temperature dependence is highlighted by the shaded red and green areas in the graph, which correspond to the 650/546 and 557/546 nm ratios, respectively.

The complex coupling between environmental and thermal effects limits the capability of traditional analytical methods to perform dual sensing using UCNPs. To disentangle the contributions of these interdependent variables, we implement a deep learning model based on a convolutional neural network (CNN) combined with a multihead self-attention (MHSA) mechanism.

Figure 2a illustrates a sketch of the network architecture. The network processes a two-channel 1D signal as input, consisting of the raw fluorescence spectra and their first derivatives. The first derivative is included to emphasize variations in peak shapes and widths, making the training process more robust against intensity fluctuations. The model consists of six 1D convolutional layers that extract local features from the spectrum, followed by an MHSA module that captures global relationships between the different emission peaks. Finally, the extracted features are fed into two parallel, fully connected branches that independently predict the sample temperature and the laser power. To prevent data leakage between closely related spectra, data sets were partitioned group-wise rather than by randomly splitting individual spectra. Test sets used contiguous excitation-power blocks held out from training, with additional tests excluding complete temperature sweeps or power bands. Metrics are averaged over five independently seeded trainings. More details on the model architecture, together with the data curation pipeline and the leakage-free training and evaluation protocol, can be found in the Supporting Information.

**Figure 2:**
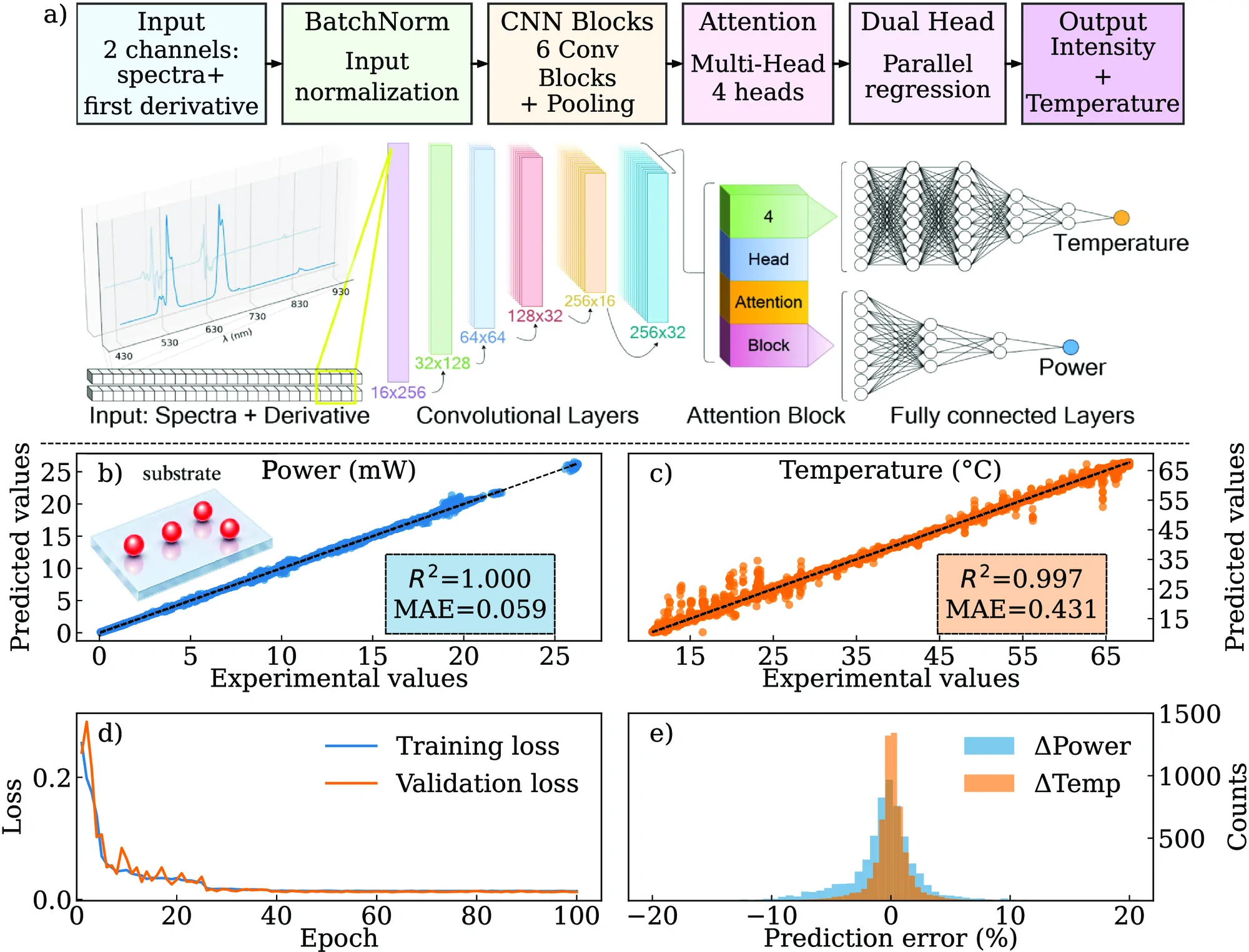
Architecture and performance of the CNN-attention dual-head model for data sets acquired from particles deposited on a substrate. (a) Schematic of the network architecture, comprising convolutional layers, multihead attention, and dual regression heads. Prediction accuracy for (b) optical power (mW) and (c) temperature (°C) with corresponding *R* ^2^ and MAE metrics. (d) Training and validation loss curves. (e) Error distribution histograms for both parameters. The model demonstrates precise multiparameter regression for optical spectroscopy applications.

The network architecture was first evaluated using the data set obtained from particles deposited on the glass substrate. The predictive performance is presented in Figure 2b–e. For this environment, the model predictions are in good agreement with the experimental measurements across all tested conditions. All the reported metrics are evaluated on test spectra belonging to excitation-power blocks entirely held out from training spectra, and correspond to the mean over independently seeded trainings (see the Supporting Information). Specifically, the neural network model achieves an *R*_*P*_^2^ of 1.000 with a mean absolute error (MAE) of 0.059 ± 0.006 mW for the laser power in Figure 2b and an *R*_*T*_^2^ of 0.997 with a MAE_*T*_ of 0.43 ± 0.20 °C for the temperature in Figure 2c. For laser powers above 2 mW, where the noise of the thermometric intensity ratio is not a limiting factor, the temperature MAE_*T*_ decreases to 0.35 ± 0.05 °C. Averaging the predictions of five independently trained models (deployable ensemble) further reduces the overall errors to 0.47 °C and 0.060 mW. The quick and smooth convergence of the training and validation loss curves, which is achieved after approximately 50–70 epochs, leads to stable results shown in Figure 2d, indicating that the network is trained efficiently without signs of overfitting. In addition, the error distributions are centered close to zero as presented in Figure 2e, confirming the stability of the predictions across the data set.

To evaluate the operational limits and robustness of the model, we performed several stress tests, see Table S1. First, we tested the effect of spectral resolution by progressively reducing the number of data points in the input spectra. The network maintained high accuracy (*R* ^2^ > 0.99 for both parameters) even with a 50% reduction in spectral resolution, defining a clear limit for faster data processing loops. Second, we artificially shifted the input spectra by a random number of pixels to simulate instrumental wavelength drifts. The convolutional layers successfully handled these shifts, preserving the predictive accuracy of the model. Such wavelength/pixel shifts emulate the use of imperfectly calibrated spectrometers. We also tested the effect of adding random Gaussian noise, representing reductions in the signal-to-noise ratio caused by detector noise, weaker emission, or shorter integration times. Across the three tests, the model retained high predictive performance under reduced spectral resolution and moderate perturbations, with degradation emerging only at stronger noise levels. These results confirm the robustness of the model against realistic instrumental variations, although substantially different particle batches or cross-lab experimental setups may still require recalibration or retraining.

To further test the versatility of our method, we trained our CNN–MHSA model on data sets acquired in water, where solvent-induced quenching typically modifies the luminescent response relative to that measured on a bare glass substrate. Despite these environmental variations, the model maintains similarly good performance in water, as summarized in Figure 3a,b. In this medium, the network yields a *R*_*P*_^2^ of 0.999 with a MAE_*P*_ of 0.148 ± 0.004 mW for the laser power, and a *R*_*T*_^2^ of 0.995 with a MAE_*T*_ of 0.59 ± 0.07 °C for the temperature (0.40 ± 0.02 °C for *P* ≥ 2 mW). The error distributions for both the substrate and water environments remain tightly centered around zero, confirming that the network successfully compensates for medium-dependent fluctuations without requiring manual parameter tuning. Notably, the temperature error in water remains essentially unchanged when test data sets, comprised of complete temperature sweeps, entire laser-power bands, or both, are fully excluded from training data sets. The errors linked to the segmented training data sets are 0.57 ± 0.05, 0.55 ± 0.08, and 0.54 ± 0.07 °C, respectively, compared to 0.55 ± 0.07 °C obtained with a full training data set. This fact demonstrates that the neural network accurately predicts parameters of spectra labeled with temperatures and powers never encountered during training and therefore learns a continuous spectrum-temperature-power relationship rather than memorizing a discrete calibration grid. Note that a similar behavior is observed for the data sets measured for ensembles of particles deposited on a glass substrate.

**Figure 3:**
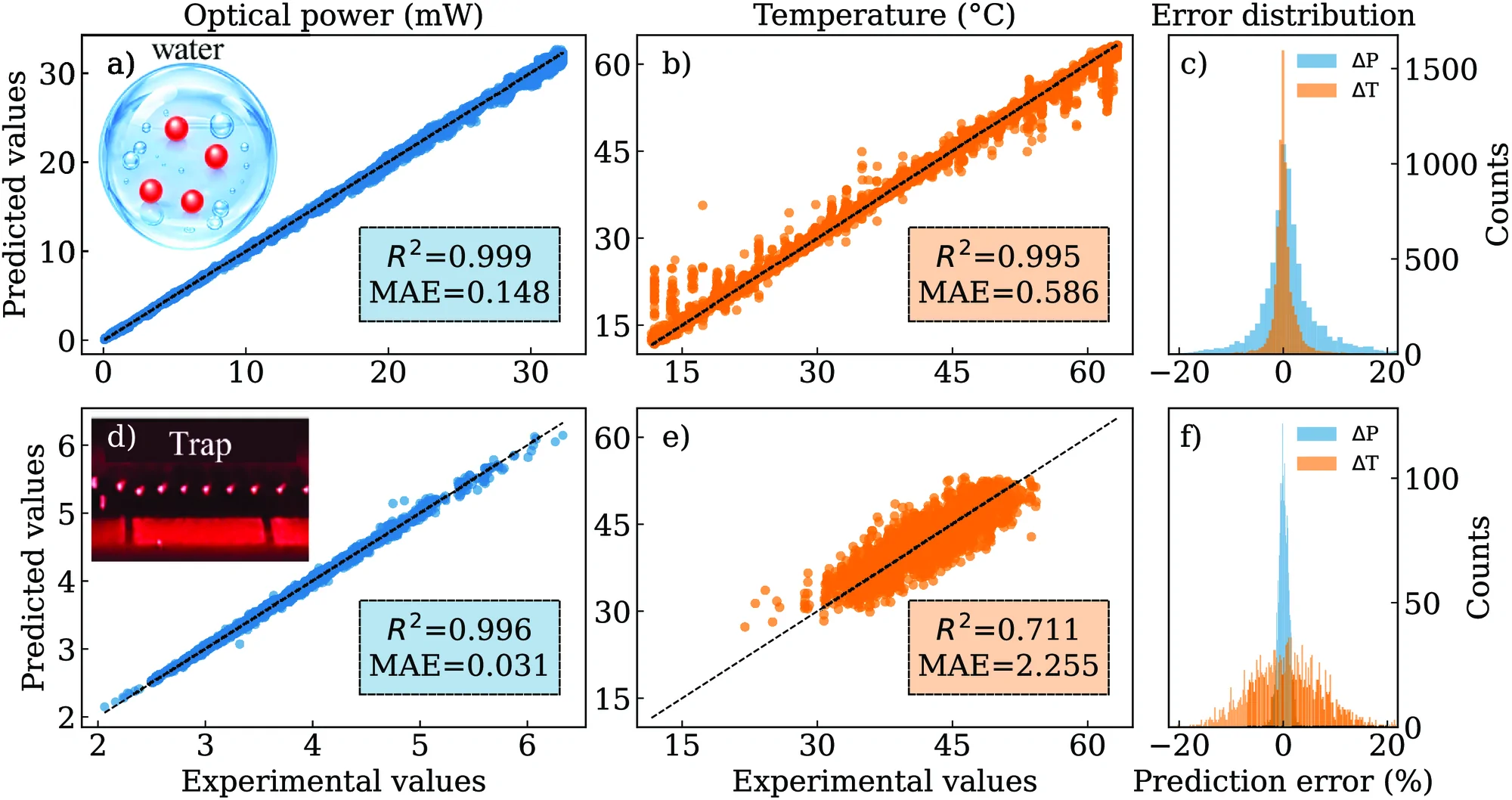
Predictive behavior of the network architecture tested for two different media, including (a–c) particles embedded in water and (d–f) trapped particles in vacuum. We plot the predicted laser powers (mW) in parts a and d, the temperature (°C) in parts b and e, and the error distribution histograms in parts c and f. The insets show the *R* ^2^ and MAE performance metrics.

Beyond data sets acquired from particle ensembles in wet and dry environments, we applied our data-driven framework to individual particles levitated in a quadrupole Paul trap. Levitated particles provide an exceptional platform for investigating the inherent mechanisms that support dual sensing because they are isolated from substrate-mediated heat conduction and chemical interactions with the surrounding media. This isolation enables the network to decode the intrinsic thermo-optical response of the material.^12^ In this configuration, particle heating is induced directly by the 980 nm excitation laser via light-induced electronic excitation followed by nonradiative electron–phonon relaxation, which is enhanced by the inefficient heat dissipation in vacuum. Consequently, temperature is less directly controlled than in the particle ensemble measurements, restricting the accessible range from approximately the unheated trap temperature up to the maximum temperature reached at the highest laser power. In addition, training the neural network on spectra acquired from individual levitated particles in vacuum provides a more stringent test of its robustness, as these measurements constitute a technically more demanding scenario with substantially lower signal levels than ensemble-based measurements. The neural-network training procedure remained unchanged, while the spectral data sets required specific preprocessing and calibration. Because no independent thermometer is available for levitated particles in vacuum, the temperature labels were obtained by combining the spectroscopic calibration of the trap measurements with the ex situ ratiometric calibrations (see the Supporting Information). These labels therefore provide a calibrated spectroscopic temperature reference rather than an independent measurement of absolute temperature. The reported metrics quantify how accurately the network retrieves the temperature from the full spectral information, while the absolute accuracy remains limited by the uncertainty of the spectroscopic calibration. The model successfully processed single-particle-level spectra, with the final predictions displayed in Figure 3d–f. The network achieved a MAE of 0.031 ± 0.005 mW for the laser power and a MAE of 2.255 ± 0.02 °C for the temperature relative to this spectroscopic reference. To provide a quantitative benchmark against a conventional full-spectrum multivariate method, we additionally trained partial least-squares (PLS) regression models^25^ using the same preprocessing and leakage-free group-wise partitioning protocol as for the CNN-MHSA model (Table S3 and Figure S5). CNN-MHSA consistently outperformed PLS for laser-power retrieval in all three environments, yielding MAEs of 0.059, 0.148, and 0.031 mW for the substrate, water, and trap data sets, respectively, compared with the best corresponding spectrum-plus-derivative PLS values of 0.412, 0.221, and 0.106 mW. CNN-MHSA also substantially improved temperature prediction for substrate and water data sets, reducing the best spectrum-plus-derivative PLS MAEs from 4.216 and 4.200 °C to 0.431 and 0.586 °C, respectively, while increasing *R* ^2^ from 0.900 and 0.867 to 0.997 and 0.995. The trap temperature predictions using PLS and CNN-MHSA provided comparable results, with MAEs of 1.922 and 2.255 °C and *R* ^2^ values of 0.771 and 0.711, respectively. This result is particularly significant because it shows that our deep-learning-driven solution remains reliable in the trapped single-particle-level regime, where weaker signals and stricter calibrations make sensing substantially more challenging.

In this work, we have developed a robust, data-driven tool built around a CNN-attention deep learning architecture to simultaneously determine local temperature and laser power using Er/Yb-doped upconverting particles. Although traditional ratiometric approaches, such as the *I*_525_/*I*_546_ ratio, and some multivariate full-spectrum methods, such as PLS, are highly reliable for temperature sensing, they do not fully decouple the complex environmental cross-talk required for dual-parameter sensing. Our proposed deep learning framework successfully overcomes this limitation by using a dual-channel input consisting of raw luminescence spectra and their first derivatives, allowing the network to capture subtle variations in emission lineshapes and peak widths.

The versatility of this tool was validated across diverse experimental conditions, demonstrating robust predictive performance in both particle ensembles deposited on glass substrates or suspended in aqueous media, and isolated single particles levitating in a vacuum trap. The network maintained reliable predictions across these scenarios without requiring manual parameter tuning or redesign of the environment-specific model. Overall, this AI-based approach provides a practical, precise, and adaptable framework for multiparameter optical sensing, advancing the use of upconverting particles as dual temperature-power sensors.

## Supplementary Material



## Notes

Additional details on the experimental methods, deep-learning architecture, data curation, training procedure, evaluation protocol, description of the optical setups, laser beam-size characterization, and spectral calibration procedures on the experimental side, and quantitative results of the stress tests applied to the proposed deep-learning method, together with the corresponding partial least-squares regression analyses (PDF)
