# Supplementary material for: Deep-Learning-Driven Dual Sensors Based on Single-Trapped Upconverting Particles
Source: Nano Lett. 2026 Oct 20;26(43):14987–92. doi: 10.1021/acs.nanolett.6c03652 (PMC13741908; doi:10.1021/acs.nanolett.6c03652)
Supplement: Supplementary file 1 [file nl6c03652_si_001.pdf]

Deep Learning-Driven Dual-Sensors Based on Single Trapped Upconverting Particles

Óscar Pérez-Benito,[†] J. Ignacio Echavarría,[¶] and Javier Hernandez-Rueda[*,¶]

†*Optics Department, Faculty of Physics, University Complutense of Madrid. Plaza de Ciencias 1, 28040 Madrid, Spain*

‡*IMDEA Nanociencia, Calle Faraday 9, Madrid, 28049, Spain*

¶*Instituto de Química Física Blas Cabrera, IQF-CSIC, Serrano 119, 28006 Madrid, Spain*

E-mail: j.hernandez.rueda@csic.es

1 Experimental Setup and Calibration

1.1 Spectroscopy and Microscopy Setup

The experimental setup employed to acquire large spectral datasets is depicted in Fig. S1 (a). First, we acquired datasets measured using ensembles of particles deposited on a substrate (borosilicate glass slide) and embedded in an aqueous suspension as shown in Fig. S1 (b), and subsequently on single trapped particles as depicted in Fig. S1 (c).

Laser power labels: Laser excitation was automated using a laser current controller (ITC4000, Thorlabs), a temperature-stabilized mount (LDM9LP, Thorlabs) and a continuous-wave diode laser at 980 nm (LP980-SA100, Thorlabs). The laser was focused on the luminescent samples by a microscope objective (Mitutoyo, Plan-Apochromat, 10×, NA=0.28).

Spectra acquisition: The upconversion signal was collected using the same objective, routed by a dichroic mirror (DMSP950, cuttoff = 950 nm, Thorlabs), filtered (FESH0900, Thorlabs) and coupled to a fiber-based spectrometer (QEPro, Ocean Insight).

Temperature labels: Temperature control in both wet and dry experiments was achieved using a thermal bath circulator connected to a hollow metal chuck, ensuring uniform thermal contact between the metal and the sample substrate or cuvette using thermal paste (RS554-311, RS pro). Real-time temperature monitoring was performed using a calibrated thermocouple (Pt100, RS pro) and a multimeter (SDM3045X, Siglent).

Levitated particle experiments: Single particle experiments were conducted using a quadrupole ion trap (i.e. Paul trap) built inside a transparent vacuum chamber.[1–3] An electrospray emitter, consisting of a 10 mL syringe with a metallic emitter connected to a high voltage 2900 V, generates a homogeneous spray known as Taylor cone[4] and introduce charged individual particles from an ethanol-based suspension into the trap. The trap consists of four gold-coated cylindrical rods (12 cm long, 3 mm radius) arranged in an 8 mm square and diagonally driven with DC (5 V) and AC (600 V, 3.0 kHz) voltages, enabling stable trapping down to 10^{-3} mbar. The polarimetry unit is used to characterize the size of trapped particles

using three probe lasers (642, 785, and 852 nm), a super-achromatic $\lambda/2$ wave plate, and a motorized rotation mount.[2] Fig. S1 (c) illustrates an optical microscopy example of confined Er/Yb-doped particles, where the bright red spots represent scattered light from the 642 nm probe beam illuminating the single trapped particles.

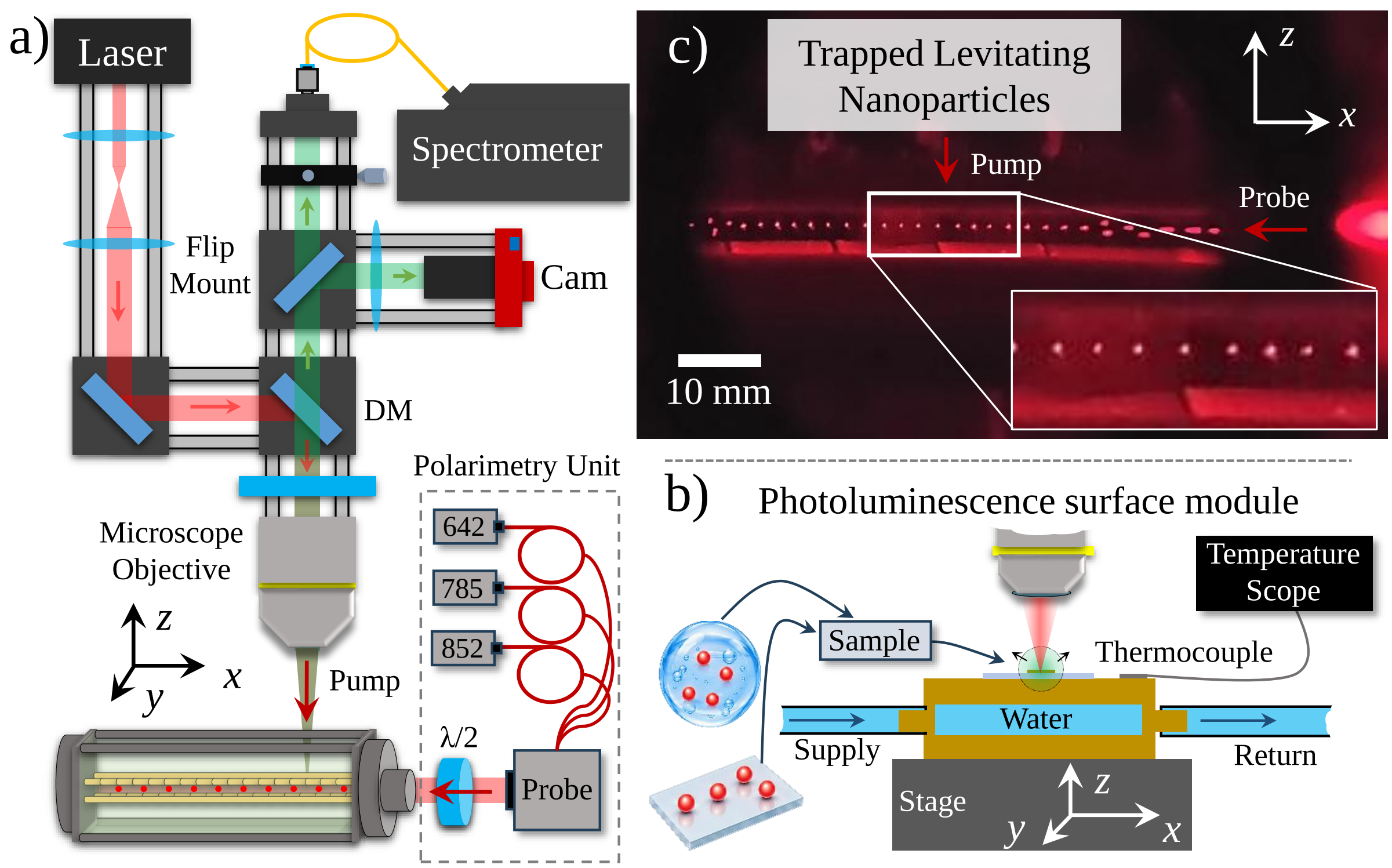

Figure S1: a) Schematic of the experimental setup in the Paul trap configuration. An excitation pump beam illuminates the UCNPs through a microscope objective, which also collects the upconversion signal for spectrometer and camera analysis. A probe beam with up to three wavelengths (642, 785, and 842 nm) and a polarimetry unit is used to characterize the size of the trapped particles. b) The surface module consists of particle ensembles deposited on a glass substrate or suspended in water inside a cuvette. These sample holders are placed on a temperature control unit connected to a thermal bath and monitored by a thermocouple. c) Optical microscopy image of UCNPs levitating in the Paul trap, illuminated by the pump beam along the perpendicular z-axis and the probe beam along the longitudinal x-axis.

1.2 Beam size characterization

1.2.1 Characterization inside the trap setup

The laser beam size in the trap was calibrated from the scattering signal of a single optically trapped particle illuminated with a continuous 980 nm laser. Fig. S2 a) illustrates the integrated scattering cross-section signal extracted from a micrograph. By laterally scanning the beam over the static particle and fitting the background-corrected scattering signal, a (1/e) beam size of (50 ± 5 μm) was obtained (see panel b) and our previously published work for more details.[1,5])

1.2.2 Beam size characterization for experiments performed out of the trap

The beam size outside the trap was measured by placing a CCD camera below the microscope objective used to focus the 980 nm laser beam. After attenuating the beam, the recorded intensity profile was fitted with a Gaussian function, yielding a beam size of 15 ± 1 μm (see Fig. S2 c).

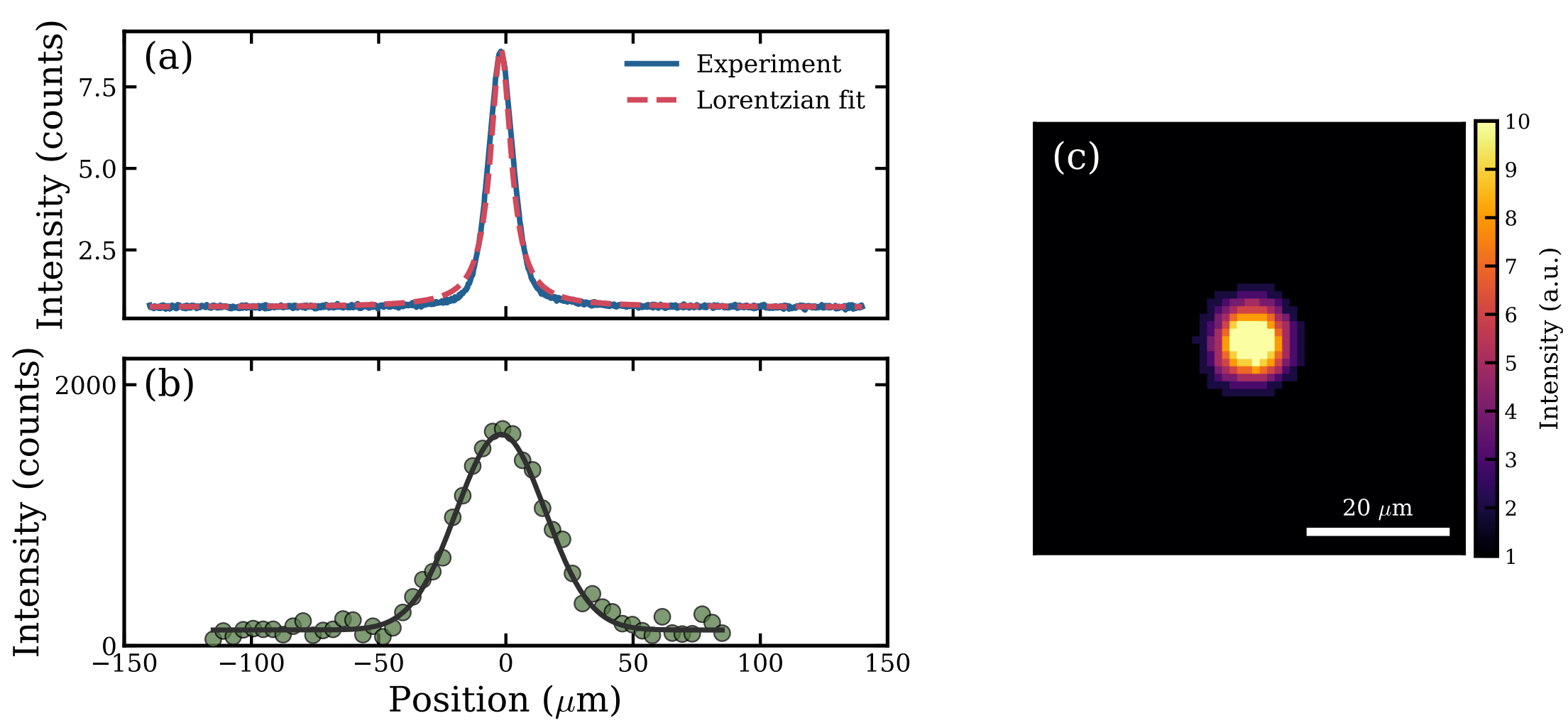

Figure S2: (a) Integrated lateral profile extracted from a microscopy image of the light scattered by a single trapped nanoparticle under 980 nm laser illumination. The red curve shows a Lorentzian fit to the experimental profile. (b) Integrated scattered-light intensity as a function of the lateral position of the laser beam relative to the fixed nanoparticle position. (c) Optical microscopy image of the laser beam profile recorded using a 4f imaging system.

2 Calibration of spectral datasets for trapped particles

We calibrated the spectral datasets measured in levitated particles and wet and dry environments, i.e. inside and outside of the trap. This allows us to make one-to-one comparisons between spectra measured in different media as well as accurately calibrate the temperature of particles inside the trap system and the laser intensity that illuminates them.

Spectral calibration: we performed an independent spectral calibration of the spectroscopy setups used to measure the particles outside and inside the trap. Both systems are optical twins, sharing the same excitation and detection geometry, with the main difference being that the Paul trap is enclosed in a transparent vacuum chamber. As a result, the light collected from trapped particles passes through the antireflection-coated glass wall of the vacuum chamber in which the trap system is embedded.

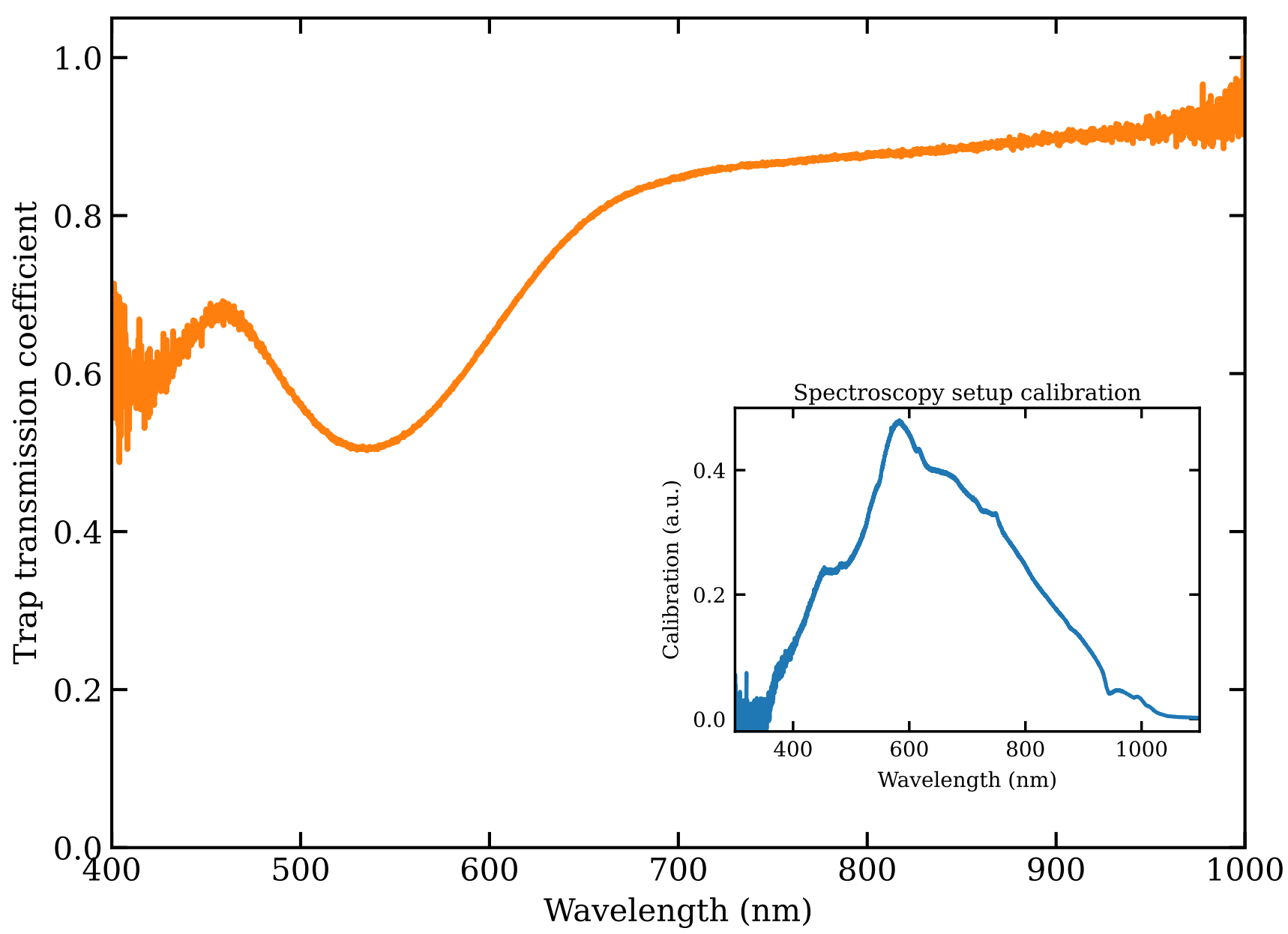

Figure S3: Transmission function of the vacuum chamber in which the trap system is embedded. The inset illustrates the transmission of the identical excitation/detection spectroscopy setups used to measure spectra in all environments.

To account for this additional wavelength-dependent transmission, the two spectroscopy configurations were calibrated separately using a white-light source with calibrated emis-

sion over the spectral range of interest (see full details in Refs.[1–3]). For each setup, the spectrum of the reference light source collected after all optical elements was divided by the calibrated lamp spectrum, yielding the corresponding spectral transmission function as shown in Fig. S1. The trap spectra were then corrected using the transmission function of the trap configuration, which includes the light passing through the chamber window.

Temperature: this procedure ensures that spectra measured in the trap can be directly compared with those acquired outside the trap. In particular, it allows the temperature retrieval of trapped particles by applying the ratiometric calibration curves obtained outside the trap that we show in Fig. 1 (c).

Laser intensity: the spectral data measured inside the trap was acquired in four different experimental runs carried out in consecutive days along 8-hour-long campaigns. The stability of the trap allows the particle to remain trapped and unchanged for a tested period of months. However, nanoparticles may slightly move $z = \pm 1\ \mu m$ with respect to their averaged most stable position along the trap z-axis, which leads to slightly different laser excitation values and introduces noise to the measurements. To minimize the impact of this experimental limitation on the CNN training procedure, we filtered out spectra that deviated a ± 10 % with respect to the averaged luminescence signal at a given laser intensity. Additionally, we removed spectra that did not contain any luminescence signal when acquired at very low laser excitation intensities.

3 Neural network architecture

The proposed Deep Learning model is a dual-head, attention-enhanced Convolutional Neural Network (CNN) implemented in PyTorch. It is designed to process dual-channel 1D input tensors containing the raw fluorescence spectrum and its first derivative, with an input length of 512 spectral data points per channel. Prior to training, each spectrum is linearly resampled to 512 points on a common wavelength grid restricted to the window containing the relevant Er^{3+} emission bands, so that all acquisition sessions share the same spectral axis. The network is sequentially structured into a convolutional feature extractor, a global attention mechanism, and task-specific regression heads as shown in the diagram in Fig. 2.

Input Normalization and Feature Extractor

The input tensor of shape `(Batch, 2, 512)` is initially normalized using a 1D Batch Normalization layer across the channel dimension. The feature extraction block consists of six sequential 1D convolutional layers that progressively increase channel dimensionality while reducing spatial resolution:

- **Layer 1:** 16 filters, kernel size 9, padding 4.
- **Layer 2:** 32 filters, kernel size 7, padding 3.
- **Layer 3:** 64 filters, kernel size 5, padding 3.
- **Layer 4:** 128 filters, kernel size 5, padding 2.
- **Layers 5 & 6:** 256 filters, kernel size 3, padding 1.

Each convolutional layer is followed by a 1D Batch Normalization layer and a ReLU activation function. Spatial downsampling is achieved using a 1D Max Pooling layer (kernel size 2) after the first five convolutional blocks. Following the final convolution, an Adaptive Average Pooling layer sets the sequence length to a fixed size of 32, yielding an output tensor of shape `(Batch, 256, 32)`.

Multi-Head Self-Attention (MHSA) Module

To capture long-range dependencies across the spectrum, the CNN output is permuted to `(Batch, 32, 256)`, treating the spatial dimension as a sequence of 32 tokens with an embedding dimension of 256. This is processed by an MHSA layer configured with 8 attention heads (`num_heads=8`, `batch_first=True`). The resulting sequence is collapsed into a single 256-dimensional feature vector per sample using mean pooling across the sequence dimension.

Dual Regression Heads

The aggregated feature vector is routed into two parallel Multi-Layer Perceptrons (MLPs) to predict particle temperature and laser intensity independently. These branches employ an alternating sequence of GELU and SiLU activation functions to model complex nonlinearities:

- **Temperature Head:** A five-layer perceptron with intermediate feature dimensions of 256, 256, 128, and 64, outputting a single scalar for temperature.
- **Intensity Head:** A three-layer perceptron with intermediate dimensions of 128 and 64, outputting a single scalar for optical power.

The outputs from both heads are concatenated along the feature dimension to form the final `(Batch, 2)` prediction tensor. A schematic representation of the model architecture is depicted in Fig. 2 (a).

4 Data curation, training and evaluation protocol

Labels and filtering. Temperatures (water, substrate) were obtained from the Pt100 readings via the Callendar–Van Dusen equation (IEC 60751), and powers from a measured current–power calibration of the diode; readings outside the calibrated ranges were discarded, never extrapolated. Trap temperature labels were taken from the spectroscopic calibration described above. Spectra were rejected if their integrated emission deviated from the monotonic emission–power trend of their session (corrupted label), if either thermometric band fell below a minimum signal-to-noise ratio or the propagated uncertainty of the I_{525}/I_{546} ratio exceeded a bound, or if they showed saturation or anomalous line shapes (principal-component reconstruction error; $<0.5\%$ of the data).

Training and evaluation. Training used AdamW (5×10^{-4}, weight decay 10^{-4}), with each spectrum weighted in the temperature loss by $1/\sigma_T^2$ (its ratio-noise-implied uncertainty), temperature-balanced sampling, seeded random generators, and retention of the best validation state. To prevent leakage between nearly identical spectra, datasets were partitioned group-wise: the test set consists of contiguous excitation-power blocks held out entirely from each temperature sweep and, for the generalization tests, complete temperature sweeps and entire power bands were additionally excluded, so that test spectra probe conditions never seen by the CNN during training. All metrics are the mean $\pm$ standard deviation over five independently seeded trainings.

5 Stress Tests: assessment of robustness against spectral and instrumental perturbations

To evaluate the robustness of the model, we applied controlled stress tests consisting of spectral-resolution reductions, wavelength shifts, added noise, and wavelength-dependent intensity-response distortions. Fig. S4 shows a representative stress test illustrating how the model performance is assessed. In this example, the model is trained using the substrate dataset with the spectral resolution reduced by 10%. Table S1 provides a broader perspective of the stress tests summarizing the averaged R^2 and MAE values for laser-power and temperature prediction at different perturbation levels for each stress test. Averaged results are calculated over three independent training runs, each evaluated on its test set, using different random seeds #1-#3. Reducing the number of spectral points reproduces measurements obtained with lower spectral resolution or reduced data acquisition and processing requirements. Artificial pixel shifts emulate wavelength-calibration errors or instrumental drift between measurements. Added Gaussian noise represents the deterioration in signal-to-noise ratio caused by detector noise, weaker emission, or shorter integration times.

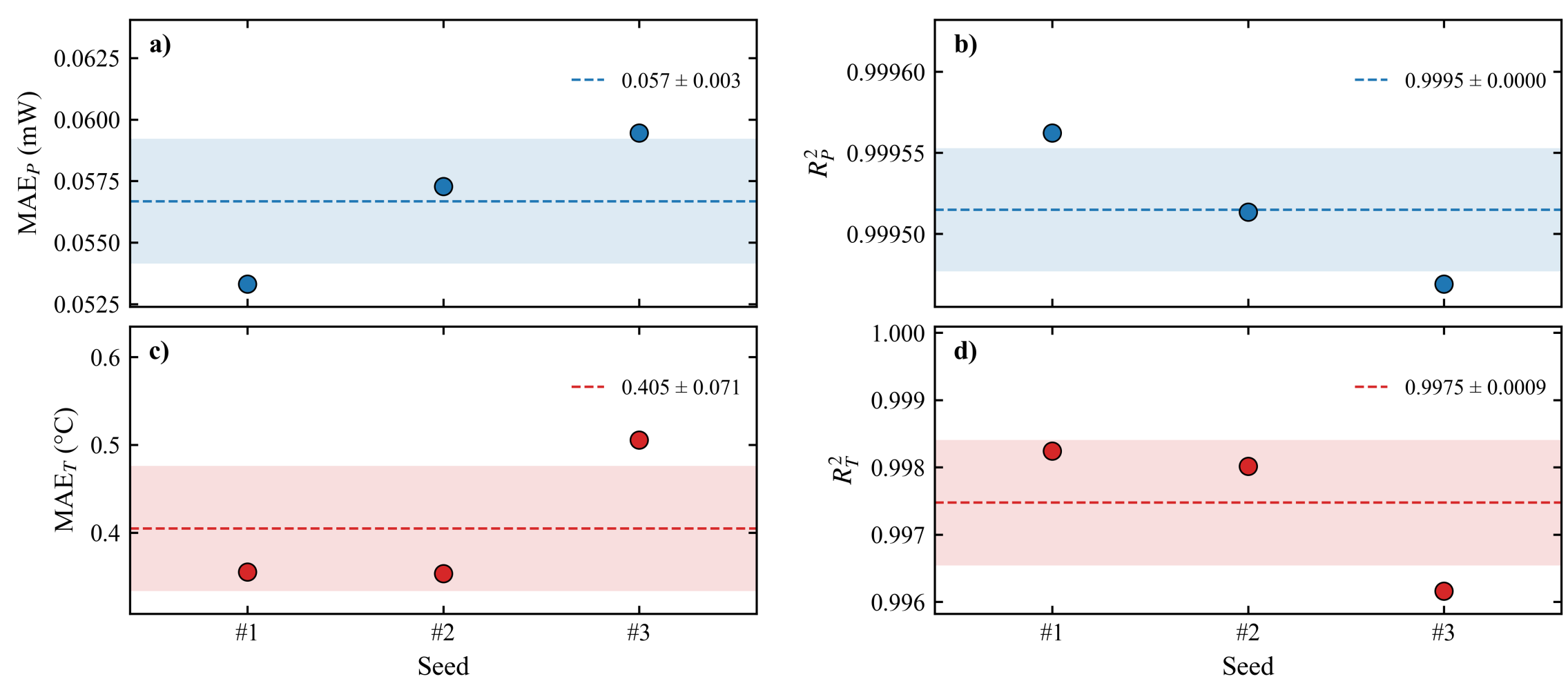

Figure S4: a) Mean absolute error and b) coefficient of determination for the excitation power prediction; c) mean absolute error and d) coefficient of determination for the temperature prediction. Dashed lines indicate the mean value over the three trainings and shaded bands the ±1 standard deviation; the corresponding values are given in each panel.

Table 1: Stress tests: quantitative evaluation of the CNN-MHSA model under controlled spectral perturbations and across the three experimental configurations. P/T denotes laser power/temperature; the corresponding MAE units are mW/°C.

	**Water**		**Substrate**		**Paul trap**	
	R^2 (P/T)	MAE (P/T)	R^2 (P/T)	MAE (P/T)	R^2 (P/T)	MAE (P/T)
**Spectral-resolution reduction**						
10% fewer spectral points	0.999/0.996	0.160/0.512	0.999/0.997	0.057/0.404	0.995/0.646	0.037/0.928
25% fewer spectral points	0.999/0.995	0.159/0.549	0.999/0.997	0.062/0.429	0.995/0.643	0.035/0.932
50% fewer spectral points	0.999/0.993	0.149/0.640	0.999/0.997	0.059/0.423	0.995/0.618	0.035/0.963
**Added spectral noise**						
Gaussian noise, $\sigma = 1\% \, I_{\max}$	0.998/0.983	0.271/1.557	0.995/0.986	0.132/1.651	0.982/0.561	0.072/1.067
Gaussian noise, $\sigma = 3\% \, I_{\max}$	0.988/0.876	0.602/4.275	0.954/0.899	0.360/4.437	0.959/0.515	0.113/1.130
Gaussian noise, $\sigma = 5\% \, I_{\max}$	0.975/0.735	0.854/6.316	0.946/0.731	0.442/7.205	0.936/0.531	0.137/1.107
**Wavelength/pixel shift**						
Random shift within ±5 pixels	0.999/0.996	0.162/0.478	0.999/0.998	0.068/0.382	0.990/0.630	0.046/0.942

Finally, wavelength-dependent intensity variations may arise from differences in optical transmission, detector quantum efficiency, and the spectral response of particles embedded in different environments. Importantly, our datasets were acquired in two experimental configurations (with and without the Paul trap) and across three distinct particle environments. The validation therefore already incorporates real spectral variations introduced by both the optical arrangement and the surrounding medium, complementing the controlled synthetic perturbations considered in the stress tests. For direct comparison, the CNN-MHSA results for the three configurations are summarized in Table S2, alongside the stress-test results in Table S1. Transfer to independent nanoparticle batches, substantially different instruments, or other laboratories may nevertheless require dedicated validation and, where necessary, recalibration or retraining.

Table 2: Summary of CNN-MHSA model training using full experimental datasets across the three experimental configurations. P/T denotes laser power/temperature; the corresponding MAE units are mW/°C.

**Experimental validation across configurations and environments**		
Particles deposited on a substrate	1.000/0.997	0.059/0.431
Particles dispersed in water	0.999/0.995	0.148/0.586
Single particle levitated in the Paul trap	0.996/0.711	0.031/2.255

6 Benchmark using partial least square regression

To provide a fair comparison with the CNN-MHSA architecture, we have trained partial least-squares regression (PLS) models using exactly the same dataset preparation and leakage-free training, validation, and test partitions employed for the neural network. All transformations were determined exclusively from the training data. We considered two input representations: the full spectrum alone and the full spectrum concatenated with its first derivative, the latter providing the same spectral information supplied to the CNN-MHSA model.

Table 3: Performance of partial least-squares regression for laser-power and temperature prediction. PLS1 denotes separate single-response models, whereas PLS2 denotes a simultaneous dual-response model.

Input	Model	Predicted variable	MAE	R^2
**SUBSTRATE**				
Spectrum	PLS1	Laser power (mW)	0.388	0.990
Spectrum	PLS1	Temperature (°C)	3.718	0.919
Spectrum	PLS2	Laser power (mW)	0.489	0.984
Spectrum	PLS2	Temperature (°C)	3.880	0.913
Spectrum + derivative	PLS1	Laser power (mW)	0.412	0.989
Spectrum + derivative	PLS1	Temperature (°C)	4.216	0.900
Spectrum + derivative	PLS2	Laser power (mW)	0.601	0.977
Spectrum + derivative	PLS2	Temperature (°C)	4.558	0.884
**WATER**				
Spectrum	PLS1	Laser power (mW)	0.231	0.999
Spectrum	PLS1	Temperature (°C)	4.105	0.873
Spectrum	PLS2	Laser power (mW)	0.313	0.998
Spectrum	PLS2	Temperature (°C)	4.152	0.870
Spectrum + derivative	PLS1	Laser power (mW)	0.221	0.999
Spectrum + derivative	PLS1	Temperature (°C)	4.200	0.867
Spectrum + derivative	PLS2	Laser power (mW)	0.379	0.997
Spectrum + derivative	PLS2	Temperature (°C)	4.219	0.866
**TRAP**				
Spectrum	PLS1	Laser power (mW)	0.107	0.953
Spectrum	PLS1	Temperature (°C)	1.928	0.770
Spectrum	PLS2	Laser power (mW)	0.109	0.952
Spectrum	PLS2	Temperature (°C)	1.930	0.770
Spectrum + derivative	PLS1	Laser power (mW)	0.106	0.954
Spectrum + derivative	PLS1	Temperature (°C)	1.923	0.771
Spectrum + derivative	PLS2	Laser power (mW)	0.108	0.953
Spectrum + derivative	PLS2	Temperature (°C)	1.922	0.771

In brief, PLS regression reduces each high-dimensional spectrum to a smaller set of latent components constructed as weighted combinations of the intensities at different wavelengths. Unlike unsupervised dimensionality-reduction methods, these components are selected according to their covariance with the reference quantities. This is suitable here because laser power and temperature both affect multiple emission bands, although in different and partially overlapping ways. The resulting components are then used to predict laser power and/or temperature. We evaluated two formulations, namely, PLS1 and PLS2 for single and dual sensing evaluation, respectively. In PLS1, two independent single-response models were trained, one for laser power and another for temperature, allowing the number of latent components to be optimized separately for each quantity. In PLS2, a single multi-response model simultaneously predicted laser power and temperature from a common set of latent components. The number of components was selected using the validation set, while the test set was reserved exclusively for the final calculation of the MAE and R^2.

The resulting MAE and R^2 values are summarized in Table S3 for the substrate, water and trap datasets, comparing separate PLS1 and simultaneous PLS2 models using either the spectra alone or the spectra concatenated with their first derivatives.

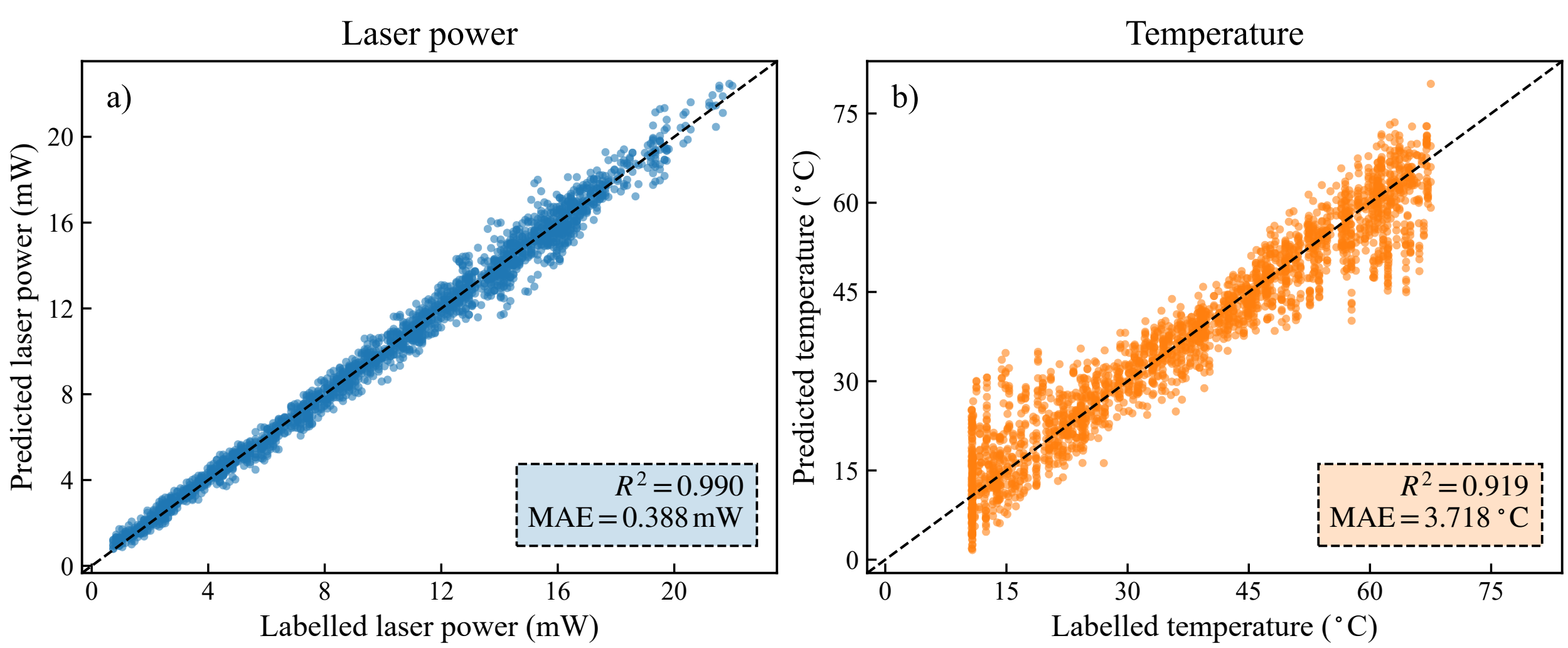

Figure S5: Predicted versus labelled laser-power and temperature values for the substrate dataset using separate spectrum-only PLS1 models.

Fig. S5 shows the predicted versus labeled a) laser-power and b) temperature values for

the substrate dataset using separate spectrum-only PLS1 models. This illustrative case provided the best overall PLS performance and therefore represents the most favorable benchmark for comparison with the CNN-MHSA model.

References

(1) Maestro, L. M.; Antón, M. A.; Cabrera-Granado, E.; Weigand, R.; Hernandez-Rueda, J. Intrinsic Optical Response of Levitating Upconverting Single Particles. *ACS Photonics* **2025**, *12*, 1783–1792.

(2) Hernandez-Rueda, J.; de Beurs, A.; van Oosten, D. Ultrafast laser ablation of trapped gold nanoparticles. *Opt. Lett.* **2019**, *44*, 3294–3297.

(3) Hernandez-Rueda, J.; Mascaró-Burguera, L.; Echavarría, J. I.; Torres-Cavanillas, R.; Coronado, E.; Pinilla-Cienfuegos, E. Photon- and pressure-controlled size modifications in single trapped spin-crossover nanoparticles. Ultrafast Phenomena and Nanophotonics XXX. 2026; p PC138940B.

(4) Taylor, G. Disintegration of water drops in an electric field. *Proceedings of the Royal Society of London. Series A, Mathematical and Physical Sciences* **1964**, 383–397.

(5) Pinilla-Cienfuegos, E.; Mascaró-Burguera, L.; Torres-Cavanillas, R.; Echavarría, J. I.; Regueiro, A.; Coronado, E.; Hernandez-Rueda, J. Unveiling Spin Transition at Single-Particle Level in Levitating Spin Crossover Nanoparticles. *ACS Nano* **2026**, *20*, 5044–5053.
